# Amorphous-Carbon-Supported Ultrasmall TiB_2_ Nanoparticles With High Catalytic Activity for Reversible Hydrogen Storage in NaAlH_4_

**DOI:** 10.3389/fchem.2020.00419

**Published:** 2020-05-15

**Authors:** Xin Zhang, Xuelian Zhang, Zhuanghe Ren, Jianjiang Hu, Mingxia Gao, Hongge Pan, Yongfeng Liu

**Affiliations:** ^1^State Key Laboratory of Silicon Materials and School of Materials Science and Engineering, Zhejiang University, Hangzhou, China; ^2^School of Chemistry and Chemical Engineering, Yantai University, Yantai, China

**Keywords:** hydrogen storage, complex hydrides, alanates, catalyst doping, cycling stability

## Abstract

In this paper, we report amorphous-carbon-supported TiB_2_ nanoparticles having sizes of 2–4 nm (nano-TiB_2_@C) as highly active catalysts for hydrogen storage in NaAlH_4_. Nano-TiB_2_@C was synthesized by a simple calcination at 550°C with Cp_2_TiCl_2_ and MgB_2_ (molar ratio of 1:1) as precursors. The addition of 7 wt% nano-TiB_2_@C reduced the onset dehydrogenation temperature of NaAlH_4_ by 100 to 75°C. A practically available hydrogen capacity of 5.04 wt% could be desorbed at 140°C within 60 min, and completely hydrogenated at 100°C within 25 min under a hydrogen pressure of 100 bar. Notably, the hydrogen capacity was almost unchanged after 20 cycles, which shows the stable cyclability, considerably higher than those of structures catalyzed by Ti halides or TiO_2_. The stable catalytic function was closely related to the *in-situ*-formed Ti–Al alloy, which considerably facilitated the dissociation and recombination of H–H and Al–H bondings.

## Introduction

Considering its high energy density, abundance, small weight, and environmental friendliness, hydrogen could provide a considerably cleaner and more sustainable society in the future (Schlapbach and Züttel, 2001). However, various challenges should be overcome to achieve the storage of hydrogen in a safe, efficient, and economic manner (Eberle et al., 2009). Metal complex hydrides composed of metal cations and complex anions (i.e., alanates, borohydrides, and amides) can store considerably more hydrogen than traditional interstitial hydrides, and thus have attracted increasing interest in recent years (Orimo et al., 2007; Jain et al., 2010). In particular, sodium alanate (NaAlH_4_) is considered a promising solid medium for hydrogen storage because of its suitable thermodynamics, relatively low desorption temperature, and good reversibility (Li et al., 2013a; Liu et al., 2018). Theoretically, NaAlH_4_ contains 7.5 wt% H_2_ which is obtained in a three-step process.

(1) $$\begin{array}{r} \text{NaAl}{\text{H}}_{\text{4}}↔\frac{1}{3}\text{N}{\text{a}}_{\text{3}}\text{Al}{\text{H}}_{\text{6}}\text{+}\frac{\text{2}}{3}\text{Al+}{\text{H}}_{\text{2}}↔\text{NaH}+\text{Al}+\frac{3}{2}{\text{H}}_{\text{2}}\\ \;↔\text{Na+Al+}2{\text{H}}_{2} \end{array}$$

However, only 5.6 wt% H_2_ from the first two steps in the above equation can be utilized for practical applications as the decomposition of NaH occurs at temperatures over 400°C (Li et al., 2013a), too high for hydrogen storage.

Several strategies have been developed to improve the hydrogen storage properties of complex hydrides, such as catalyst doping (Frankcombe, 2012; Liu et al., 2018), cations substituting (Jain et al., 2010; Fang et al., 2011; Mo and Jiang, 2018), fabrication of reactive composites (Vajo et al., 2005; Ding et al., 2015; Mustafa et al., 2018), and nanostructuring (Ding and Shaw, 2019; Ding et al., 2019a, 2020). Recently, Shaw's group have developed a ball milling process with aerosol spraying to fabricate a nanocomposite of LiBH_4_ and MgH_2_ and successfully achieved the dual-tuning effects of the thermodynamics and kinetics of LiBH_4_ (Ding et al., 2019b). Regarding NaAlH_4_, numerous studies have shown that the addition of appropriate catalysts is crucial for a reduction in its hydrogen storage operation temperatures (Liu et al., 2018). In 1997, for the first time, Bogdanović and Schwickardi have reported a reduction (higher than 80°C) in desorption temperature of NaAlH_4_ by doping 2 mol% β-TiCl_3_ (Bogdanović and Schwickardi, 1997). Since then, various Ti-based additives have been introduced into NaAlH_4_, particularly Ti halides and oxides, the most investigated catalysts (Frankcombe, 2012). Using TiF_3_, Wang et al. have reported a release of H_2_ above 2.5 wt% at 120°C (Wang et al., 2005; Kang et al., 2007). Lee et al. observed superior catalytic activity for nano-TiO_2_ over TiCl_3_ because a nano-TiO_2_-containing NaAlH_4_ has released ~3 wt% H_2_ at 150°C within 10 min while only 2.5 wt% H_2_ has been released from a TiCl_3_-doped sample (Lee et al., 2008). Moreover, complete hydrogen release from NaAlH_4_ was realized with a nano-TiO_2_/C composite catalyst at 140°C within 30 min, with up to an H_2_ capacity of 4.5 wt% (Liu et al., 2016).

However, the introduction of high-electronegativity anions, such as O, F, Cl, and Br, has reduced the effective hydrogen capacity because these anions tend to combine with Na and/or Al and consume the active components of hydrogen storage. Thus, methods to simultaneously achieve low dehydrogenation temperatures and high practical hydrogen capacities are required. In this regard, Ti-based compounds composed of low-electronegativity anions, such as TiN, TiC, and TiB_2_, have come in sight for their catalytic activities. A reversible storage capacity of 4.9 wt% H_2_ has been demonstrated within 16 cycles by doping TiN into NaAlH_4_ (Bogdanovic et al., 2003). A NaAlH_4_-2%TiN mixture has exhibited a capacity above 5 wt% H_2_ at 250°C (Li et al., 2013b). A rod-shaped nano-TiN@C-N composite has reduced the hydrogen desorption temperature to 140°C with an H_2_ capacity of 4.9 wt% (Zhang et al., 2018). Relatively high hydrogen capacity was also obtained for TiB_2_-doped NaAlH_4_ (Li et al., 2012a,b; Liu et al., 2014). However, only a limited reduction in dehydrogenation temperature has been attained so far. Further increase in the catalytic effectiveness of TiB_2_ is still desired.

In this study, we synthesized an amorphous-carbon-supported nanoparticulate TiB_2_ (nano-TiB_2_@C) by calcining a mixture of Cp_2_TiCl_2_ and MgB_2_. The fabricated nano-TiB_2_ has a size of 2–4 nm and exhibited a remarkable catalytic activity for the hydrogen storage reaction of NaAlH_4_. A reduction in onset dehydrogenation temperature higher than 100°C was achieved using a 7 wt% nano-TiB_2_@C, which provided a practically available hydrogen capacity of 5.04 wt%. Furthermore, almost no capacity loss was observed within 20 cycles, which is superior to the performances of reported TiB_2_-modified samples. The chemical states of nano-TiB_2_@C and corresponding catalytic mechanisms were analyzed.

## Experimental

### Fabrication of Materials

All reagents and solvents were commercially available and used as received without further purification. Nano-TiB_2_@C was synthesized by calcining a mixture of titanocene dichloride (Cp_2_TiCl_2_, 97%, Aladdin) and MgB_2_ (97%, Alfa Aesar) with a molar ratio of 1:1 under an Ar atmosphere at 550°C for 2 h. The resultant powders were collected, washed twice with tetrahydrofuran (THF) to remove the byproduct MgCl_2_, and dried under vacuum at 150°C for 12 h to yield the nano-TiB_2_@C composite.

Nano-TiB_2_@C was mixed with NaAlH_4_ (hydrogen storage grade, Sigma Aldrich) to evaluate its catalytic effectiveness. The weight percent of nano-TiB_2_@C with respect to NaAlH_4_ was designed to be 0, 1, 3, 5, 7, or 9 wt%. The sample mixing was carried out using a QM-3SP4 planetary ball mill under a hydrogen pressure of 50 bar. Approximately 1 g of the mixture was loaded into the milling jar inside an MBRAUN glovebox (Germany) filled with pure argon (H_2_O and O_2_ < 1 part per million). The ball-to-sample weight ratio was ~120:1. The mill rotated for 0.3 h in one direction, paused for 0.1 h, and then rotated in the reverse direction for another 0.3 h.

### Characterization

The dehydrogenation was qualitatively evaluated using a home-developed temperature-programmed desorption (TPD) system attached to an online gas chromatograph (GC). The sample (~40 mg) was heated from room temperature to 400°C at 2°C min^−1^ with pure Ar as a carrier gas at a flow rate of 20 ml min^−1^. Quantitative dehydrogenation and hydrogenation properties were evaluated using a home-developed Sieverts-type apparatus. Isothermal and non-isothermal measurements were performed on samples having masses of ~60 mg. In the non-isothermal experiments, a heating rate of 2°C min^−1^ and primary vacuum (~10^−3^ Torr) were used for dehydrogenation, while a heating rate of 1°C min^−1^ and hydrogen pressure of 100 bar were used for hydrogenation. In the isothermal measurements, the samples were rapidly heated to a desired temperature and kept at that temperature during the entire test.

X-ray diffraction (XRD, X'Pert Pro, Rigaku, Japan) with Cu K_α_ radiation (40 kV, 40 mA) was carried out for identification of phases. XRD patterns were acquired in a 2θ range of 10–90° with step increments of 0.05°. The sample powders were sealed in a custom-designed container with a window covered by Scotch tape to prevent air and moisture contaminations. An elemental analysis was performed using a Vario MICRO cube element analyzer (Elementer, Germany) to quantify the contents of Ti, B, and C elements. Scanning electron microscopy (SEM, Hitachi, S-4800) and transmission electron microscopy (TEM, FEI, Tecnai G2 F20 S-TWIN) were used for morphology observations. The distributions of elemental Ti, B, and C were identified using an energy-dispersive X-ray spectrometer (EDS) attached to the Tecnai G2 F20 S-TWIN TEM facility. X-ray photoelectron spectroscopy (XPS) analyses were carried out using a Kratos AXIS Ultra DLD spectrometer with a monochromatic Al K_α_ X-ray source at a base pressure of 6.8 × 10^−9^ Torr. Fitting was carried out suing the XPSPEAK41 software.

## Results and Discussions

**Figure 1:**
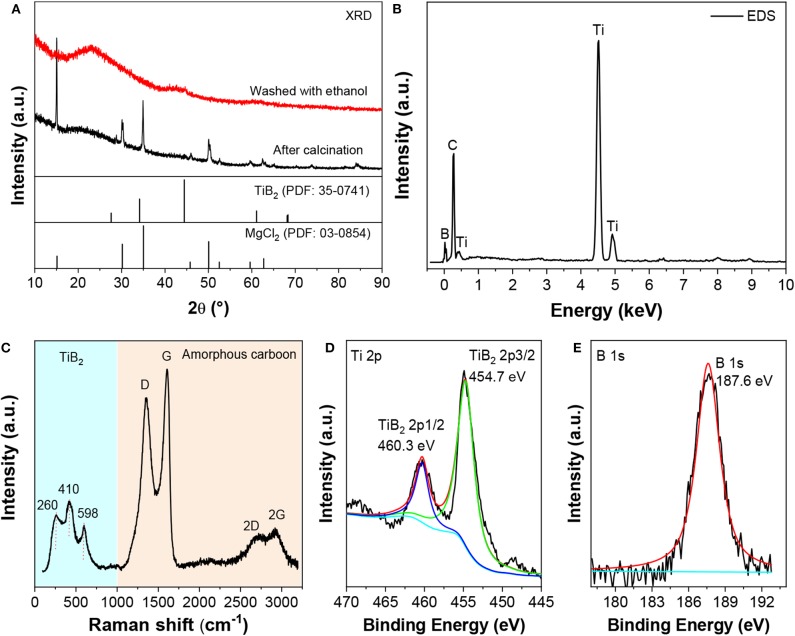
XRD patterns **(A)**, EDS profile **(B)**, Raman spectrum **(C)**, and Ti 2p **(D)** and B 1s **(E)** XPS spectra of nano-TiB_2_@C.

The structure and composition of the fabricated nano-TiB_**2**_@C were analyzed by XRD, EDS and XPS. The results are shown in Figure 1. The calcinated sample exhibited the diffraction peaks of MgCl_2_ (Figure 1A). After the washing with THF, only a broad bump at 44.4° (2θ) was observed in the XRD profile. The low and broad peaks indicate low crystallization and/or small particle/grain sizes. Ti, B, and C were detected by EDS (Figure 1B). An element analysis shows that their weight ratio was ~29:14:57, corresponding to a molar ratio of Ti and B of 1:2. A Raman spectrum analysis indicates that the elemental C was in its amorphous form (Figure 1C). Three characteristic peaks of TiB_2_ were observed at 260, 410, and 598 cm^−1^ (Bača and Stelzer, 2008). The high-resolution XPS spectra (Figures 1D,E) show characteristic peaks of the Ti–B bonding at binding energies of 460.3/454.7 eV for Ti 2p and 187.6 eV for B 1s (Ding J. C. et al., 2019). Combining the XRD, EDS, and XPS results, we believe that TiB_2_ and amorphous carbon were formed by calcining the mixture of Cp_2_TiCl_2_ and MgB_2_.

**Figure 2:**
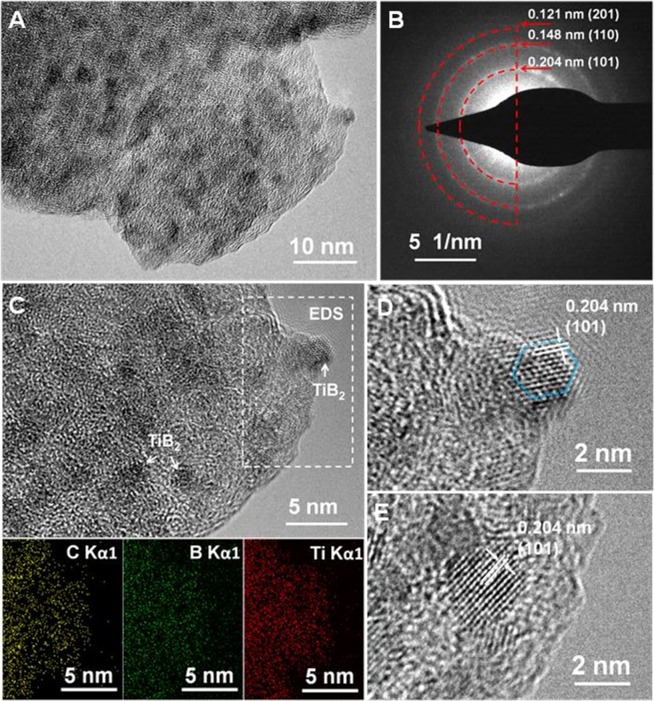
TEM image **(A)**, SAED pattern **(B)**, TEM image and corresponding EDS maps of Ti, B, and C elements **(C)**, and HRTEM images **(D,E)** of nano-TiB_2_@C.

TEM, HRTEM, EDS mapping, and selected-area electron diffraction (SAED) analyses were carried out. The TEM image (Figure 2A) shows a large number of black nanoparticles distributed in a gray matrix. The EDS mapping (Figure 2B) reveals that the small nanoparticles consisted of Ti and B, while the gray matrix was mainly C. The SAED pattern indicates (101), (110), and (201) planes assigned to TiB_2_ (Figure 2C). The HRTEM images (Figures 2D,E) indicated an interplanar spacing of 0.204 nm, corresponding to the interplanar distance of the (101) planes of TiB_2_. The particle sizes of the TiB_2_ were ~2–4 nm, part of which displayed clearly hexagon structures (Figure 2D). These results reveal nanoparticulate TiB_2_ well-dispersed in the amorphous carbon matrix.

**Figure 3:**
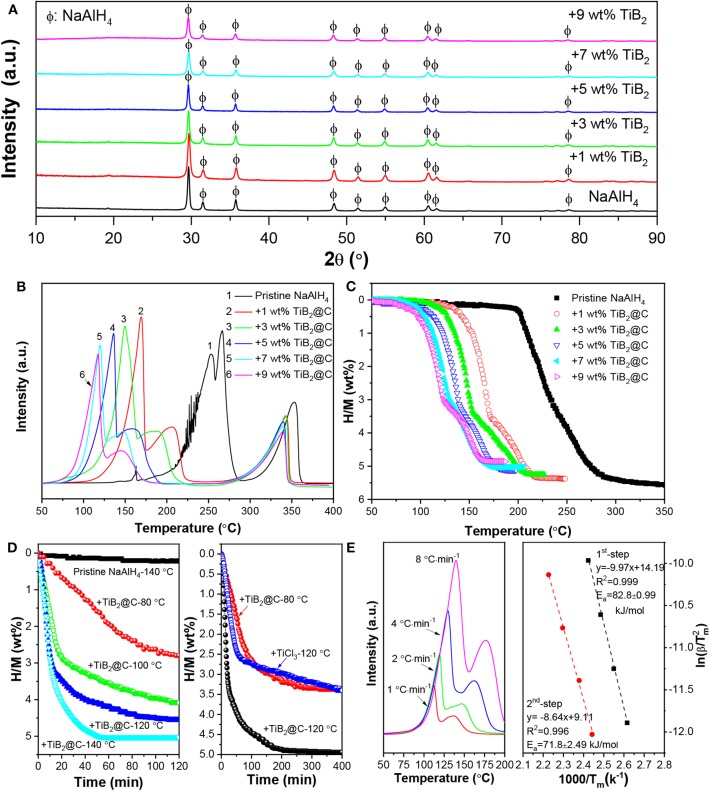
XRD patterns **(A)** and TPD **(B)** and volumetric release curves **(C)** of NaAlH_4_ with and without nano-TiB_2_@C. Isothermal dehydrogenation curves **(D)** and Kissinger's plots **(E)** of NaAlH_4_-7 wt %-nano-TiB_2_@C sample.

**Figure 4:**
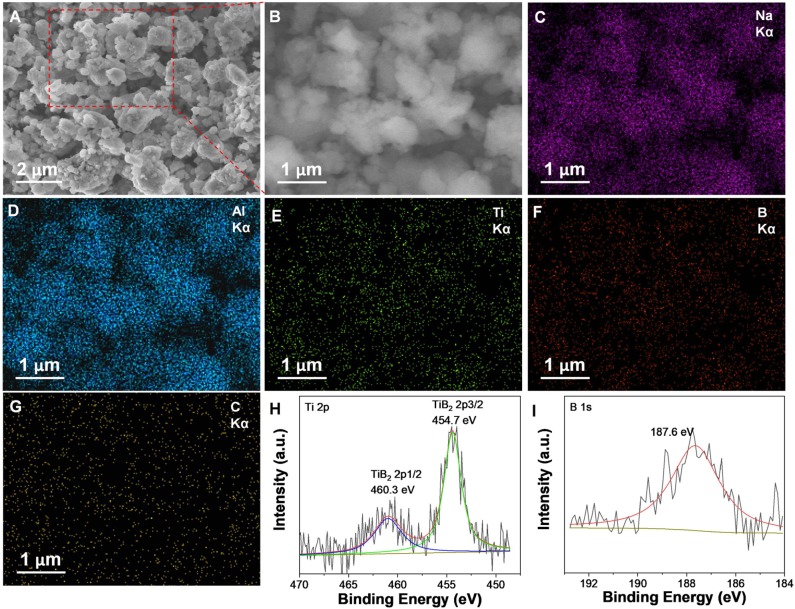
SEM images **(A,B)**, corresponding EDS maps of Na **(C)**, Al **(D)**, Ti **(E)**, B **(F)**, and C **(G)** elements, and Ti 2p **(H)**, and B 1s **(I)** XPS spectra of the nano-TiB_2_@C-containing sample.

The resultant nano-TiB_2_@C was mixed with NaAlH_4_ by ball milling to test its catalytic effectiveness. After the ball milling, all samples exhibited very similar XRD patterns, as shown in Figure 3A. With the increase in amount of nano-TiB_2_@C, the diffraction intensities of the NaAlH_4_ phase slightly decreased. The SEM images reveal irregular solid particles with sizes of 200 nm−2 μm for 7 wt% nano-TiB_2_@C-containing sample (Figures 4A,B). The EDS mapping results indicate relatively homogenous distribution of Ti, B, and C on NaAlH_4_ particles (Figures 4C–G). Although no Ti-, B-, and C-containing phases were identified by XRD, possibly owing to their amorphous forms (Figure 3A), the XPS results show Ti 2p and B 1s spectra assigned to TiB_2_ with binding energies of 454.7/460.3 and 187.6 eV (Figures 4H,I), respectively, indicating the presence of TiB_2_ upon the mixing with NaAlH_4_. We therefore believe that nano-TiB_2_@C was uniformly distributed into the NaAlH_4_ matrix.

The nano-TiB_2_@C-containing NaAlH_4_ samples were subjected to TPD and volumetric measurements for qualitative and quantitative characterization of their hydrogen storage performances. Three dehydrogenation peaks were observed in the TPD curves of all samples (Figure 3B), corresponding to the three-step decomposition process of NaAlH_4_ with the increase in temperature (Equation 1). Furthermore, the nano-TiB_2_@C-containing samples exhibited considerable low-temperature shifts. Upon the addition of 1 wt% nano-TiB_2_@C, the dehydrogenation peak associated to the first dehydrogenation step (Equation 1) shifted from 255 to 169°C, a reduction of 86°C. The increase in nano-TiB_2_@C content up to 7 wt% further reduced the start and end temperatures of the first-step decomposition to 75 and 118°C, 100 and 137°C lower than those of the pristine NaAlH_4_, respectively. In addition to the slightly reduced peak intensities, the shape of TPD curve was almost unchanged with the further increase in content of nano-TiB_2_@C. This result indicates that 7 wt% nano-TiB_2_@C was optimal for the improvements in hydrogen storage performance of NaAlH_4_.

Figure 3C shows volumetric release curves of the nano-TiB_2_@C-modified samples. As expected, the 7 wt%-nano-TiB_2_@C-containing sample exhibited the optimal dehydrogenation properties in terms of dehydrogenation temperature and hydrogen capacity in this study. Approximately 5.04 wt% of hydrogen was released in the temperature range of 75–175°C, which is remarkably superior to the performance of previously reported TiB_2_-doped NaAlH_4_ and other transition-metal-catalyzed NaAlH_4_ structures (Table 1) (Wang et al., 2005; Lee et al., 2008; Fan et al., 2009; Naik et al., 2009; Li et al., 2012a,b, 2013b; Liu et al., 2014). In the isothermal test, the same amount of hydrogen (5.04 wt%) was released within 50 min at 140°C (Figure 3D). In contrast, <1 wt% of hydrogen was released from the pristine NaAlH_4_ under the same conditions. At 120°C, the 7 wt%-nano-TiB_2_@C-containing sample could desorb 4 wt% H_2_ within 30 min, showing a much faster dehydrogenation kinetics than those of well-studied TiCl_3_-modified NaAlH_4_ (Figure 3D) (Bogdanović and Schwickardi, 1997; Naik et al., 2009). Even at 80°C, ~3.5 wt% H_2_ could be desorbed, though a period of 400 min was required. These dehydrogenation kinetics outperform those of other TiB_2_-doped NaAlH_4_, which released only 2.79 wt% under the same conditions (Li et al., 2012b). Using the Kissinger's method (Kissinger, 1957), the apparent activation energies (*E*_*a*_) were determined to be ~82.8 and 71.8 kJ/mol for the first and second dehydrogenation of the 7 wt%-nano-TiB_2_@C-containing sample, respectively (Figure 3E), which are ~ 40% lower than those of pristine NaAlH_4_ (Zhang et al., 2016) and is responsible for the remarkably reduced dehydrogenation temperatures.

**Table 1:** Comparison of desorption performances of catalyst-doped NaAlH_4_ samples.

**Catalyst**	**Non-isothermal desorption**			**Isothermal desorption**			**References**
	**On-set temperature (**°**C)**	**Terminal temperature (**°**C)**	**Capacity (wt%)**	**Temperature (**°**C)**	**Time (min)**	**H_**2**_ release (wt%)**	
TiB_2_	150	300	5.3	150	900	3	Li et al., 2012a
TiB_2_	75	250	4.9	120	400	3.2	Li et al., 2012b
TiCl_3_	100	210	4.7	150	240	4.5	Lee et al., 2008
Nano-TiO_2_	125	225	5	150	240	4.7	
TiF_3_	–	–	–	120	240	3.75	Wang et al., 2005
TiN	120	220	5.37	190	600	5.37	Li et al., 2013b
TiC	–	–	–	165	480	4.5	Fan et al., 2009
VCl_3_	160	250	4	–	–	–	Naik et al., 2009
ScCl_3_	150	225	4.3	–	–	–	Naik et al., 2009
This work	75	175	5.04	140	50	5.04	

**Figure 5:**
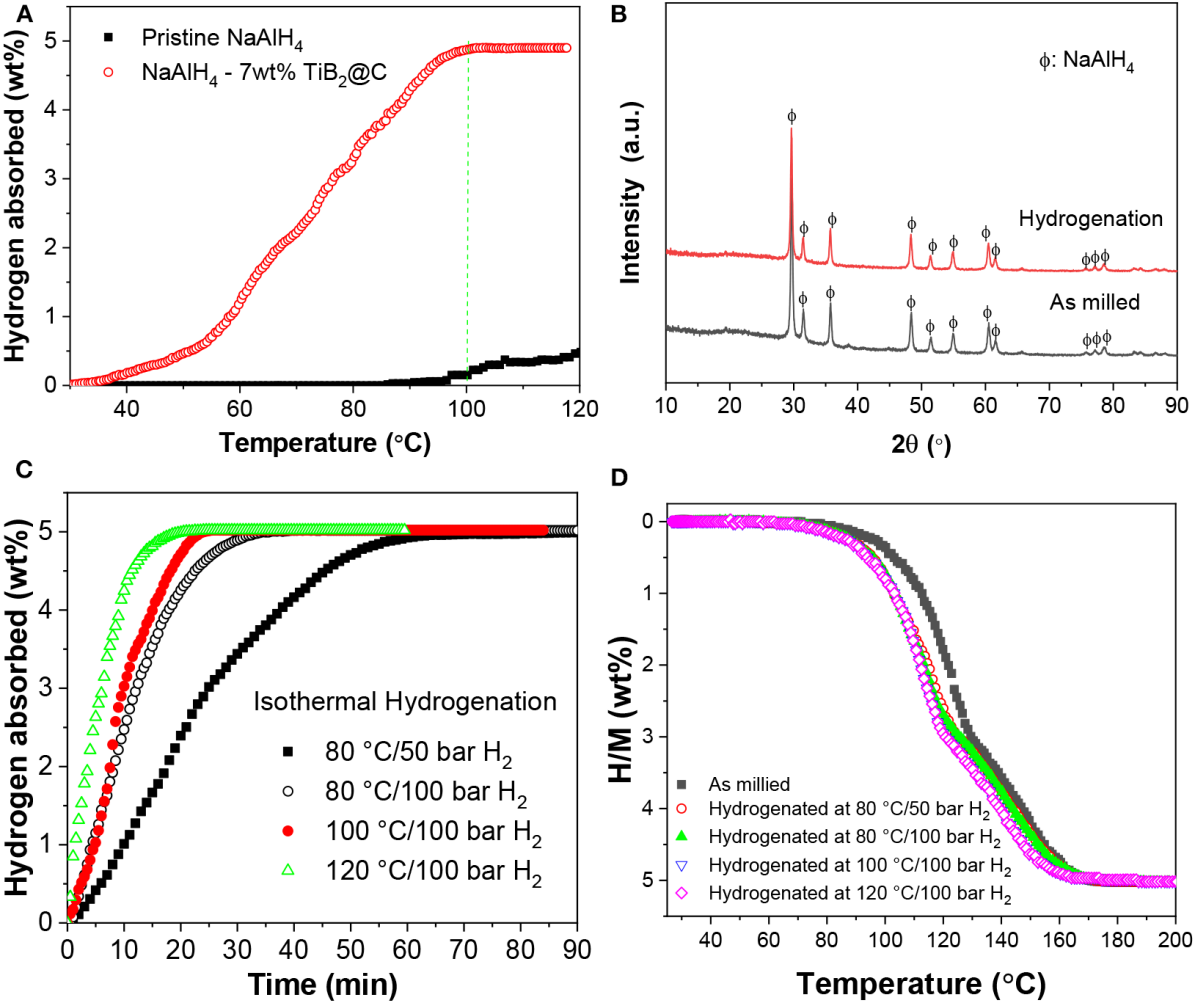
Nonisothermal hydrogenation curves **(A)** and XRD patterns of the hydrogenated products **(B)**. Isothermal hydrogenation (**C)** and redehydrogenation curves **(D)** of NaAlH_4_-7 wt%-nano-TiB_2_@C.

The dehydrogenated samples were re-hydrogenated under an H_2_ pressure of 100 bar. As shown in Figure 5A, the sample containing 7 wt%-nano-TiB_2_@C started to absorb hydrogen at a temperature of 30°C, 70°C lower than that of the sample without doping. The hydrogenation was completed at 100°C in the non-isothermal test. The XRD analysis indicates that NaAlH_4_ was formed after the full hydrogenation (Figure 5B). Isothermal hydrogenation under an H_2_ pressure of 100 bar reveals that ~5.02 wt% of hydrogen recharged into the dehydrogenated 7 wt%-nano-TiB_2_@C-containing sample within 35 min at 80°C, which provided full hydrogenation (Figure 5C). At 120°C, only 20 min were required to complete full hydrogenation, which shows the considerably faster kinetics. The follow-up dehydrogenation repeatedly resulted an H_2_ capacity of 5.02 wt% (Figure 5D), which shows the good reversibility.

**Figure 6:**
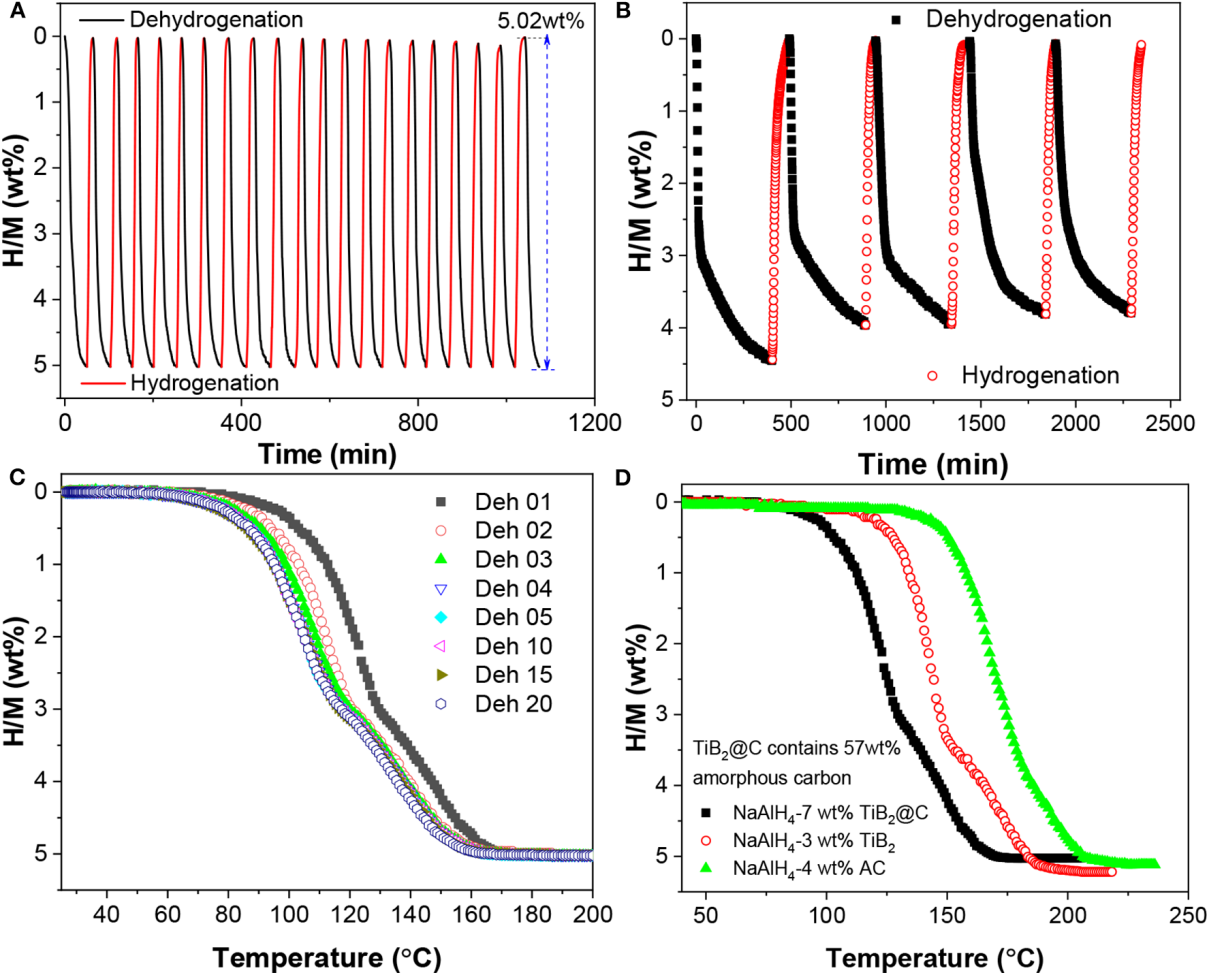
Isothermal dehydrogenation/hydrogenation cycle curves of NaAlH_4_ doped with 7 wt% nano-TiB_2_@C **(A)** and 7 wt% TiCl_3_ **(B)**, non-isothermal dehydrogenation/hydrogenation cycle curves of NaAlH_4_-7 wt% nano-TiB_2_@C **(C)**, and volumetric release curves of NaAlH_4_ samples doped with TiB_2_@C, TiB_2_, and active carbon **(D)**.

Figure 6A shows the cyclic stability of NaAlH_4_-7 wt%-nano-TiB_2_@C. Here, the dehydrogenation was conducted at 140°C in vacuum, while the hydrogenation took place at 100°C under an H_2_ pressure of 100 bar. After 20 cycles, the available hydrogen capacity still remained at 5.02 wt%, which shows the stable recyclability. This cycling stability is superior to that of the well-studied TiCl_3_-catalyzed NaAlH_4_ (Figure 6B). In addition, a small but continuous reduction in onset dehydrogenation temperature was observed in the first four cycles (Figure 6C), which reflected the activation. This might correlate to some changes in catalytic active species during the initial de-/hydrogenation cycles. Further comparison reveals that hydrogen release from the nano-TiB_2_@C-containing NaAlH_4_ occurred at lower temperatures than those of samples with either TiB_2_ or active carbon (AC) (Figure 6D), which shows the synergistic effect of TiB_2_ and C, similarly to the previous observation for NaAlH_4_ co-catalyzed by NbF_5_ and single-walled carbon nanotubes (Mao et al., 2011, 2012).

**Figure 7:**
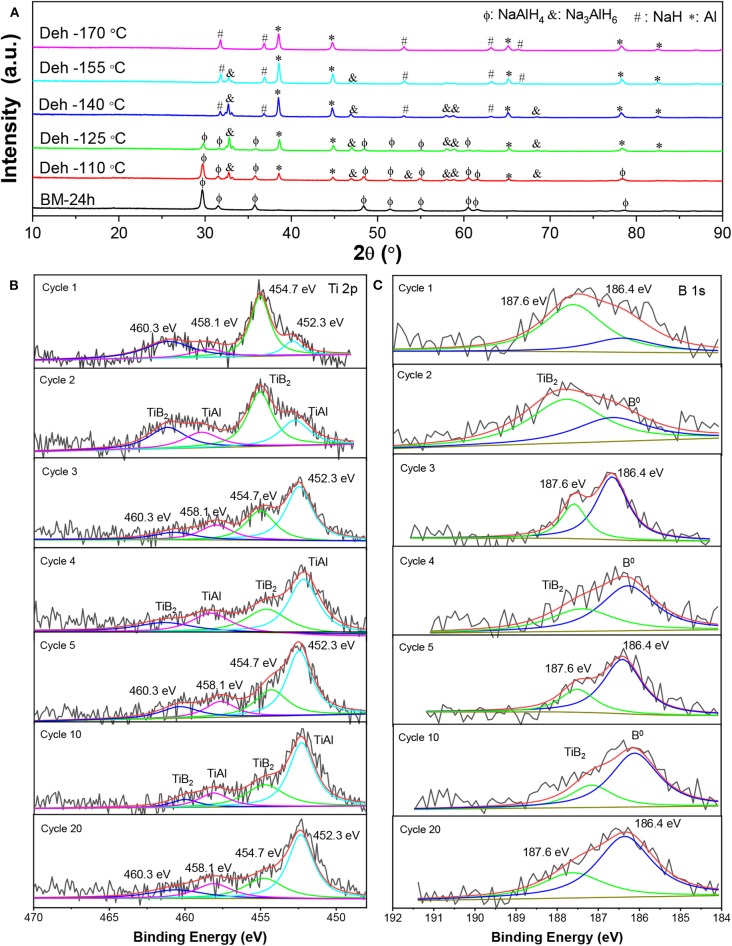
XRD patterns as a function of the dehydrogenation temperature **(A)** and high resolution Ti 2p **(B)** and B 1s **(C)** XPS spectra of the nano-TiB_2_@C-containing sample after different numbers of dehydrogenation cycles.

**Figure 8:**
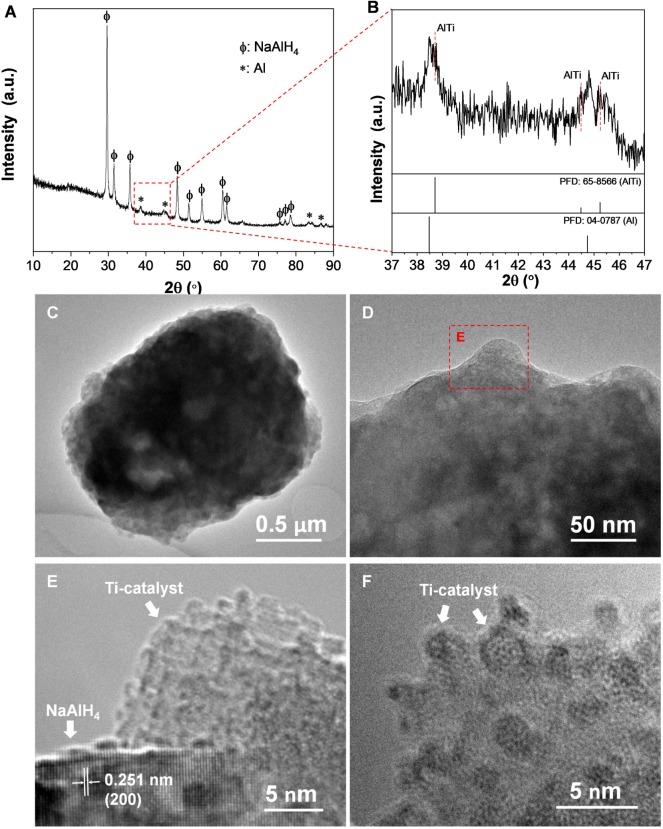
XRD patterns **(A)**, enlarged view of the XRD patterns in the range of 37–47° **(B)**, TEM images **(C,D)** and HRTEM images (with different magnifications) **(E,F)** of nano-TiB_2_@C-containing NaAlH_4_ sample after 20 cycles.

Figure 7A shows XRD patterns of the dehydrogenated samples containing 7 wt% nano-TiB_2_@C as a function of the temperature. The results indicate that with the increase in temperature, NaAlH_4_ initially decomposed to Na_3_AlH_6_ and Al (110–140°C), which then led to the formation of NaH and Al (155–175°C) with the hydrogen release. We therefore believe that the presence of nano-TiB_2_@C did not alter the dehydrogenation course of NaAlH_4_. Notably, no Ti-containing species was identified by the XRD profiles. Subsequently, high-resolution Ti 2p and B 1s XPS spectra were acquired to understand the chemical states of TiB_2_ (Figures 7B,C). Upon cycling, a 2p_3/2_-2p_1/2_ spin–orbit doublet at 452.3/458.1 eV emerged, and then became dominant in the Ti 2p XPS spectra (Figure 7B), which can be assigned to Ti–Al bonding (Mencer et al., 1991). Further XRD analysis confirms the presence of an AlTi alloy (Figures 8A,B). The AlTi alloy surface is favorable for the dissociation and recombination of H–H and Al–H bondings (Frankcombe, 2012; Liu et al., 2018). In contrast, the XPS peaks of Ti at 454.7/460.3 eV were largely reduced. On the other hand, the characteristic XPS peak of B^0^ at 186.4 eV was also detected, which gradually increased in the initial four cycles. Thus, upon the de-/hydrogenation cycling, TiB_2_ was gradually converted to Ti–Al and B, possibly reacting with NaAlH_4_. This is crucial for the continuous reduction in dehydrogenation temperature of the nano-TiB_2_@C-modified sample in the initial four cycles. The newly formed Ti–Al and B remained stable in the following cycles, which led to a good cycling stability, as shown in Figure 6A.

The nano-TiB_2_@C-containing sample subjected to 20 cycles was used for a TEM observation. As shown in Figures 8C–F, a large number of Ti catalyst nanoparticles (sizes < 5 nm) were dispersed on the surface of the NaAlH_4_ particle. Therefore, we believe that ultrasmall particles of TiB_2_ as precursors facilitated the formation of ultrafine dispersive Ti–Al active species. The dispersive distribution of Ti catalysts provided the high catalytic activity for hydrogen storage in NaAlH_4_, particularly for long-term cycling (Figure 4D).

## Conclusions

In this work, nano-TiB_2_@C below 5 nm was synthesized. Remarkable reduction in dehydrogenation and hydrognaiton temperatures was observed when adding 7 wt% nano-TiB_2_@C to NaAlH_4_. The hydorgen desorption started at a temperature of 75°C, which is lowered by 100°C compared to the pristine NaAlH_4_. A practical hydrogen capacity of 5.04 wt% was determined, which was released within 50 min at 140°C. The rehydrogenation occurred at 30°C under a hydrogen pressure of 100 ba, and was completed at 100°C. Notably, no capacity loss was observed in the 20 cycles. During the initial de-/hydrogenation cycling, TiB_2_ presumably reacted with NaAlH_4_ and was converted to AlTi alloy and zero-valence B, which were well-dispersed on the surface of the NaAlH_4_ particles, and consequently contributed to the high stable catalytic activity. These findings could facilitate the practical use of NaAlH_4_ as a high-capacity reversible hydrogen storage medium.

## Data Availability Statement

All datasets generated for this study are included in the article/supplementary material.

## Author Contributions

XiZ and YL conceived the study and designed the experiments. XiZ, XuZ, and ZR carried out the material syntheses, characterization, and measurements. XiZ, YL, JH, MG, and HP analyzed the data. XiZ, JH, and YL wrote the manuscript.

## Conflict of Interest

The authors declare that the research was conducted in the absence of any commercial or financial relationships that could be construed as a potential conflict of interest.
